# Domestic Summary

**Published:** 1839-07-27

**Authors:** 


### DOMESTIC SUMMARY.

### THE LATE NAVAL EXAMINATION.

*Navy Department*, July 19th, 1839.

The Board of Naval Surgeons, recently convened in Philadelphia, terminated its proceedings on the 12th inst.

The following assistant surgeons were examined, and found qualified for promotion: D. C. McLeod, to retain his original position on the register, next below J. A. Lockwood; Ninian Pinckney, Robert T. Barry, Geo. W. Peete, to retain their relative position on the register.

The following candidates for admission into the Navy, were passed in the order, as to relative merit, here stated:—1. John O'Connor Barclay. 2. J. B. Gould. 3. Chas. H. Wheelwright. 4. R. W. Jefferey. 5. Thos. M. Potter. 6. Wm. A. Nelson. 7. G. G. Wilson. 8. J. H. Wright. 9. N. T. H. Moore. 10. Jos. Hopkinson. 11. Jno. Thornley. 12. Danl. L. Bryan.

